# Assessing 3 Outbreak Detection Algorithms in an Electronic Syndromic Surveillance System in a Resource-Limited Setting

**DOI:** 10.3201/eid2609.191315

**Published:** 2020-09

**Authors:** Emily Alsentzer, Sarah-Blythe Ballard, Joan Neyra, Delphis M. Vera, Victor B. Osorio, Jose Quispe, David L. Blazes, Luis Loayza

**Affiliations:** Massachusetts Institute of Technology, Cambridge, Massachusetts, USA (E. Alsentzer);; Uniformed Services University of the Health Sciences, Bethesda, Maryland, USA (E. Alsentzer, S.B. Ballard, D.L. Blazes);; Johns Hopkins Bloomberg School of Public Health, Baltimore, Maryland, USA (S.-B. Ballard);; Naval Medical Research Unit No. 6, Callao, Peru (S.B. Ballard, D.M. Vera, V.B. Osorio, J. Quispe);; Marina de Guerra del Perú, Callao (L. Loayza)

**Keywords:** outbreak detection algorithms, syndromic surveillance, acute diarrheal disease, resource-limited setting, enteric infections

## Abstract

We evaluated the performance of X-bar chart, exponentially weighted moving average, and C3 cumulative sums aberration detection algorithms for acute diarrheal disease syndromic surveillance at naval sites in Peru during 2007–2011. The 3 algorithms’ detection sensitivity was 100%, specificity was 97%–99%, and positive predictive value was 27%–46%.

Syndromic surveillance uses prediagnostic health-related data to signal probable outbreaks warranting public health response (*1*). Alerta and Vigila are internet-based syndromic surveillance systems successively implemented by Peru’s navy (*2*–*4*). Among other disease syndromes, individual cases of acute diarrheal disease (ADD) are self-reported to healthcare workers at sites providing care for service members, dependents, and civilian employees. We assessed the performance of 3 ADD aberration detection algorithms in this resource-limited setting: X-bar chart, exponentially weighted moving average (EWMA), and Early Aberration Reporting System (EARS) C3 cumulative sums (CUSUM) models (*5*–*9*).

## The Study

We defined ADD as >3 loose stools within 24 hours lasting <14 days, epidemic threshold as the incidence of cases in excess of normal for a given period, outbreak as the detection of ADD incidence above the epidemic threshold, true outbreak as an outbreak identified by the system with confirmation by trained field personnel (e.g., enteropathogen isolation), and epidemiological silence as a period during which no cases were reported. We performed descriptive analysis of ADD cases by using data from all reporting sites during 2007–2011, then conducted subsequent analyses by using data on nonbloody ADD. We calculated ADD incidence from weekly reports from Alerta and Vigila during 2007–2011 for naval bases with population denominator data available. We compared the number of site-weeks during which >1 ADD case was reported on shore-based sites with those of sea-based sites and nonbloody ADD incidence in summer with incidence in nonsummer months by using a Mann-Whitney test.

Sites with <4 months of epidemiologic silence during 2007–2011 (60 months) were included for outbreak detection analysis. We aggregated nonbloody ADD case counts by epidemiologic week and performed a timeseries analysis by using X-bar chart, EWMA, and modified EARS C3 CUSUM aberration detection algorithms to flag potential outbreak weeks (*8*). Algorithm details are provided in the Appendix. 

To account for seasonal variability, nonbloody ADD cases for each week during 2009–2011 (36 months) were compared with an 8-week sliding historical baseline calculated from the current and previous 2 years (*10*,*11*). We excluded signals from weeks with <5 cases to minimize false signals associated with epidemiologic silence. Because of ADD’s <1-week incubation period, we did not consider buffer intervals, except when implementing the C3 CUSUM-like algorithm, which sums positive differences in cases from the mean for the past 3 periods. We optimized X-bar k, EWMA k and λ, and CUSUM k and h by exploring X-bar and EWMA k and CUSUM h values ranging from 2 to 6, CUSUM k values 1 to 3.5, and λ values 0.25 to 0.5, choosing parameters to maximize specificity and positive predictive value (PPV) while maintaining perfect sensitivity in predicting outbreaks at a randomly selected site (Policlínico Naval Ancón) (*7*). We calculated algorithm sensitivity, specificity, and PPV and compared each model’s performance by using pairwise exact McNemar tests with Bonferroni correction, using data from 5 sites capable of confirming true outbreaks through epidemiologic links (517 site-weeks). We performed all analyses in R version 3.6.3 (https://cran.r-project.org/bin/windows/base/old/3.6.3); p values <0.05 were considered statistically significant. 

**Figure 1:**
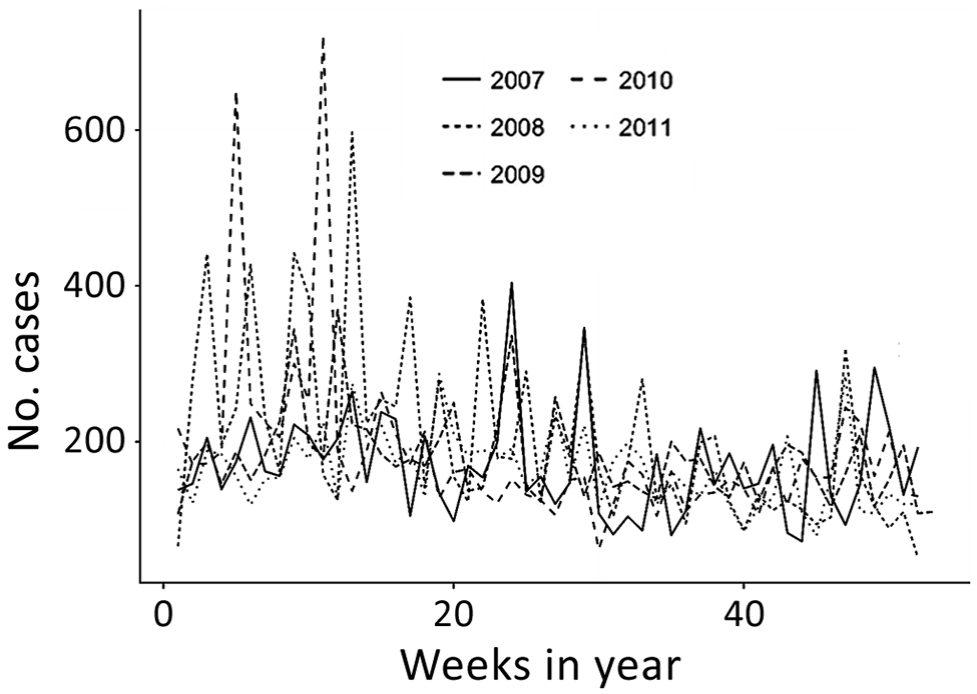
Epidemic curve for nonbloody acute diarrheal disease cases, by week, captured by the Alerta and Vigila Systems, Peru, 2007–2011.

During 2007–2011, a total of 144 sites reported 48,409 ADD cases, 98% of which were nonbloody. A total of 8,860 nonbloody cases were reported from 91 sites in 2007, 10,775 cases from 101 sites in 2008, 9,347 cases from 107 sites in 2009, 9,698 cases from 120 sites in 2010, and 8,588 cases from 118 sites in 2011. Of all these cases, 87% occurred in persons >5 years of age, 9% in children 1–4 years of age, and 4% in children <1 years of age. During 2007–2011, nonbloody ADD incidence peaked in 2008 at 305.2 cases/1,000 population (Figure 1). Seasonal incidence was higher during Peru’s summer months (median 14.3 cases/1,000 population), January through March, compared with other months (median 12.5 cases/1,000 population; p = 0.0003).

The median proportion of weeks during which a site reported >1 ADD case was 19% (interquartile range [IQR] 5%–44%), and the median length of epidemiologic silence was 58 weeks (IQR 13–147 weeks). We observed no statistically significant difference in the proportion of weeks with >1 ADD case between sea-based and shore-based sites (p = 0.55).

We established the aberration detection algorithm by using 6,962 site-weeks of data from the 45 sites with <4 months of epidemiologic silence during 2007–2011 (Table). These reporting sites consisted of 15 ships and 30 land bases from 20 districts within 14 provinces. Site populations ranged from 35 to 10,000 (median 210, IQR 93.75–450). Algorithm parameter sensitivity analysis yielded optimal results when X-bar k = 4.5, EWMA k = 4 and λ = 0.25, and CUSUM k = 1.5 and h = 3 (Appendix Figures 1–3).

**Table:** Nonbloody acute diarrheal disease case count and incidence, 2007–2011, and true outbreak detection data and algorithm performance, 2009–2011 for the 45 naval surveillance sites in Peru, analyzed by using X-bar chart, exponentially weighted moving average, and Early Aberration Reporting System C3 cumulative sums models*

Surveillance site	Total cases, 2007–2011	Average cases/week/1,000 population, 2007–2011	Date of true outbreak detection, case count, incidence, 2009–2011	Average outbreak detection algorithm performance, 2009–2011
BAP Aguirre	393	13.45	2009 Jan 24, 25 cases, 109/1,000 population	Sensitivity 100%, specificity 97.4%, PPV 20.0%
BAP Bolognesi	334	11.04	‡	‡
BAP Carvajal	581	14.56	‡	‡
BAP Eten	129	27.95	‡	‡
BAP Grau	887	9.04	‡	‡
BAP Mariátegui	490	16.64	‡	‡
BAP Montero	321	12.39	‡	‡
BAP Paita	185	†	‡	‡
BAP Palacios	1,118	27.16	‡	‡
BAP Pisagua	224	55.04		
BAP Pisco	283	†		
BAP Quiñones	289	9.17		
BAP Sánchez Carrión	156	39.90	‡	‡
BAP Velarde	114	36.54	2011 Sep 5, 12 cases, 250 cases/1,000 population	Sensitivity 100%, specificity 100%, PPV 100%
BAP Villavicencio	251	9.20	‡	‡
Base Aeronaval	991	4.19	‡	‡
Base Naval Chimbote	349	4.90	‡	‡
Base Naval Nanay	596	4.54	‡	‡
Base Naval San Juan	313	25.74	‡	‡
BCT Aguaytia	214	39.81	‡	‡
BCT Contamana	261	†		
BCT Huipoca	154	46.81	‡	‡
BCT San Alejandro	118	26.22	‡	‡
Capitanía Puerto Mollendo	171	19.52	‡	‡
Capitanía Puerto Puno	246	26.74	‡	‡
Centro Instrucción Técnica Naval	829	3.48	‡	‡
Clínica Naval de Iquitos	3,269	1.84	‡	‡
Comandancia Primera Zona Naval	244	21.02	‡	‡
Comandancia Tercera Zona Naval	625	21.48	2010 Aug 14, 31 cases, 207 cases/1,000 population	Sensitivity 100%, specificity 99.4%, PPV, 50.0%
Dirección de Capitanías y Guardacostas	293	5.77	‡	‡
Dirección de Hidrografía y Navegación	385	8.09	‡	‡
Escuela Naval	988	4.84	‡	‡
Estación Naval Isla San Lorenzo	449	24.34	‡	‡
Estación Naval Mollendo	343	6.42	‡	‡
Estación Naval Paita	796	8.93	‡	‡
Estación Naval Pucallpa	2,098	15.22	‡	‡
Estación Naval Submarinos	734	16.33	2010 Feb 8, 21 cases, 100 cases/1,000 population	Sensitivity 100%, specificity 99.1%, PPV 42.9%
Estación Naval de la Comandancia General	1,260	†	‡	‡
Hospital Base Naval del Callao	4,132	1.62	‡	‡
Policlínico Naval Ancón	2,019	5.24	2010 Feb 11, 33 cases 18 cases/1,000 population; 2010 Feb 20, 22 cases, 12 cases/1,000 population	Sensitivity 100%, specificity 98.1%, PPV 40.0%
Policlínico Naval San Borja	2,892	134.51	‡	‡
Posta Naval de Ventanilla	1,110	113.15	‡	‡
Villa Naval de Tumbes–El Salto	710	†	‡	‡

*BAP, Buque de la Armada Peruana (Peru Navy ship); BCT, Base Contraterrorist (Counter-terrorist base); PPV, positive predictive value. †No denominator data available. ‡True outbreak data not available because of lack of on-site capability to establish epidemiologic link.

**Figure 2:**
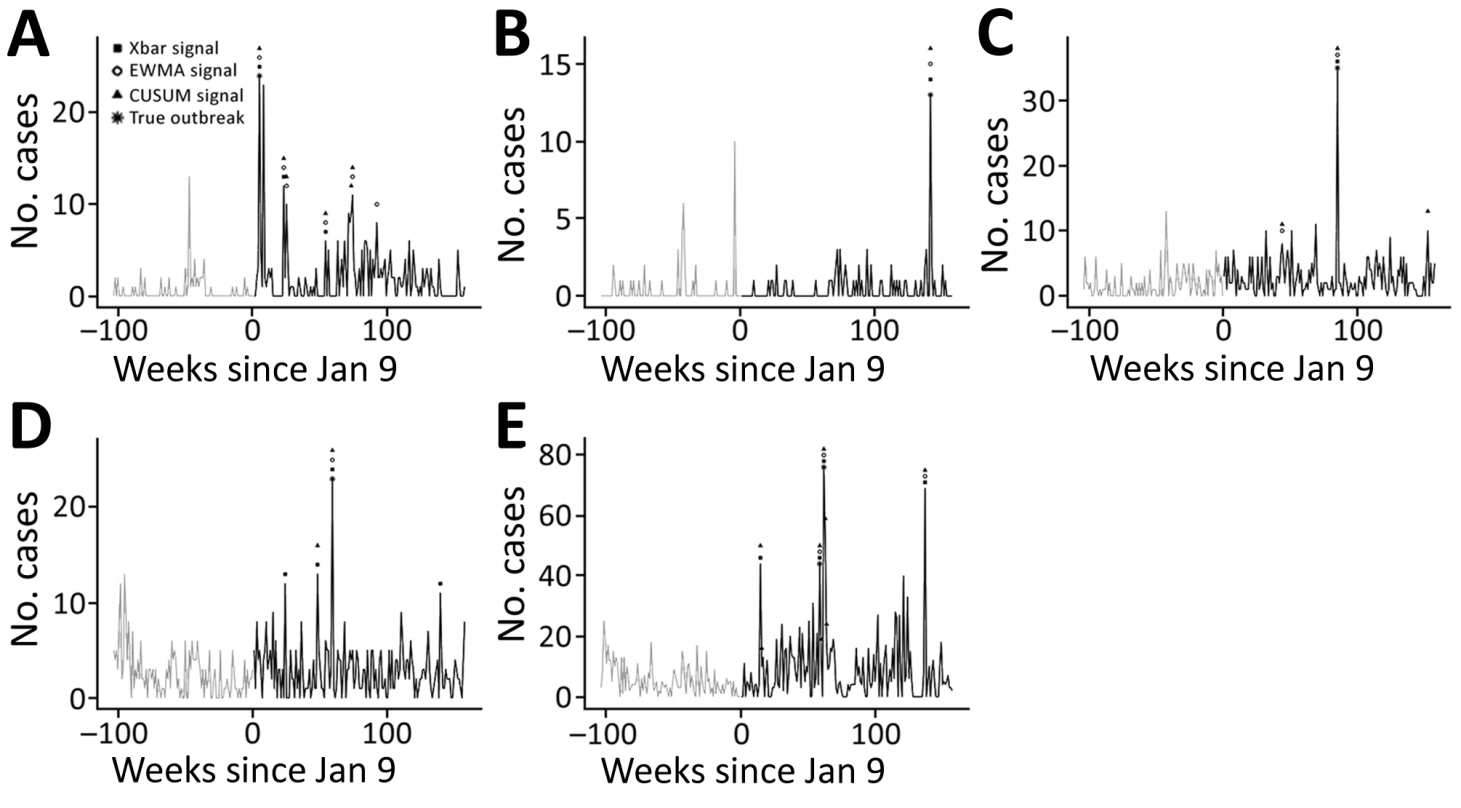
Epidemic curves for nonbloody acute diarrheal disease over time, demonstrating outbreaks identified by the Alerta and Vigila Systems, by identifying algorithm and surveillance site, Peru, 2009-2011. A) Buque de la Armada Peruana Aguirre; B) BAP Velarde; C) Comandancia Tercera Zona Naval; D) Estación Naval Submarinos; and E) Policlínico Naval Ancón. Shapes correspond with identifying algorithms; asterisks indicate outbreaks confirmed through epidemiologic links (i.e., true positives). CUSUM, C3 cumulative sums; EWMA, exponentially weighted moving average.

We estimated algorithm sensitivity, specificity, and PPV by categorizing 785 weeks of data into positive and negative outbreak weeks and comparing each algorithm’s outbreak predictions with data from the 5 sites capable of confirming true outbreaks during 2009–2011 (Figure 2). X-bar produced 13 signals, EWMA 13 signals, and CUSUM 20 signals. Six true outbreaks occurred (Table); all were detected by X-bar, EWMA, and CUSUM algorithms, corresponding to 100% sensitivity for each algorithm. Algorithm specificity across the 5 sites was 99.1% for X-bar, 99.1% for EWMA, and 98.2% for CUSUM. PPV was 46.2% for X-bar, 46.2% for EWMA, and 30% for CUSUM. X-bar and EWMA each produced 7 false-positives, and CUSUM produced 14 false-positives. The performance differences were not statistically significant.

## Conclusions

X-bar, EWMA, and C3 CUSUM aberration detection algorithms identified all ADD outbreaks during 2009–2011, and approximately one third to one half of algorithm outbreak signals corresponded to true outbreaks. These findings suggest that these algorithms can usefully inform outbreak asset deployment, particularly in resource-limited settings.

Overall, X-bar and EWMA performed marginally better than CUSUM (PPV 46% vs. 30%). CUSUM frequently produced false-positives in the weeks after large outbreaks (e.g., after the 76-case outbreak at Policlínico Naval Ancón) (Table). X-bar’s successful detection of ADD in the context of weekly reporting schedules and short disease incubation periods is consistent with its design, which favors detection of events lasting 1 epidemiologic period. In contrast, EWMA and CUSUM were designed for earlier detection of consecutive small baseline deviations (*12*). Whereas EWMA can be tuned to favor shorter outbreaks through its weighting parameter (λ), the C3 CUSUM algorithm is more rigid. In the context of weekly ADD reporting, EWMA and CUSUM algorithm performance might improve by counting consecutive weeks with outbreak signals as a single alert.

Our study has limitations, including the number of sites capable of confirming outbreaks, which reduced the statistical power to detect differences between algorithms, and the inability to distinguish a lack of reporting versus a lack of ADD cases to report during periods of epidemiologic silence. Combined, these factors limited the evaluation to 3,925 site-weeks of observation, reducing the reliability of algorithm parameter estimates. Furthermore, model parameters were established on only 1 of the 5 evaluation sites; a larger development set would better optimize model parameters while avoiding overfitting.

Characterizing algorithm parameter tradeoffs might aid system capability alignment with health priorities. Lower detection thresholds are advantageous for high-risk diseases with distinct syndromes, such as cholera, Ebola, and Middle East respiratory syndrome. Conversely, changing parameters, such as changing EWMA’s weighting parameter (λ), can affect algorithm PPV (increasing to >60% during sensitivity analysis). Context-focused exploration could inform which parameters should be tuned to improve PPV, sensitivity, or specificity. Because smaller populations and stable disease baselines improve algorithm performance, tuning algorithm parameters for specific sites during implementation by using historical data might improve overall system performance, as might periodic evaluation of model assumptions, parameter tuning, and model performance.

Appendix Additional information about assessing 3 outbreak detection algorithms in an electronic syndromic surveillance system in a resource-limited setting
