# Supplementary material for: Assessing 3 Outbreak Detection Algorithms in an Electronic Syndromic Surveillance System in a Resource-Limited Setting
Source: Emerg Infect Dis. 2020 Sep;26(9):2196–200. doi: 10.3201/eid2609.191315 (PMC7454051; doi:10.3201/eid2609.191315)
Supplement: Appendix — Additional information about assessing 3 outbreak detection algorithms in an electronic syndromic surveillance system in a resource-limited setting [file 19-1315-Techapp-s1.pdf]

Article DOI: https://doi.org/10.3201/eid2609.19-1315

Assessing 3 Outbreak Detection Algorithms in an Electronic Syndromic Surveillance System in a Resource-Limited Setting

Appendix

Introduction to X-bar Chart, EWMA, and CUSUM Algorithms, Adapted from R. Fricker (2013) (*1*).

X-bar chart, EWMA, and the CUSUM family algorithms are common methods for outbreak detection with limited historical outbreak data. All algorithms calculate an expected number of cases for a given time period (t) and produce outbreak signals if the algorithm statistic surpass the Upper Control Limit (UCL).

X-bar: The algorithm statistic for X-bar chart is the number of expected cases for a given time period, $n_{expected}$, which is defined as the average number of cases occurring in the sliding baseline (*2*). The UCL is defined as

$$n_{expected}+k*\sigma_t$$

where σ_t is the standard deviation of cases in the current baseline period and k is a hyperparameter.

EWMA: The algorithm statistic for EWMA is the exponentially weighted average of all previous cases, including the current time period, giving greater weight to most recent data. The EWMA for a given week (t) is defined as

$$\lambda * n_t + (1-\lambda)EWMA_{t-1}$$

where n_t is the number of cases for the current week and λ is a weighting factor that determines how far back in time the weighting is distributed, with only the most recent weeks having influence for higher values of λ. The UCL is defined as

$$\mu_t + k\sigma_t * \sqrt{(\lambda/(2-\lambda)}$$

where μ_t is the mean number of cases in the current baseline period, σ_t is the standard deviation of cases in the current baseline, k is a hyperparameter, and λ is as defined above.

CUSUM: CUSUM methods detect successive positive deviations from baseline means. The Centers for Disease Control's Early Aberration Reporting System (EARS) employs CUSUM-based C1, C2, and C3 algorithms for bioterrorism surveillance. The algorithm statistic for one time period (t) is defined as

$$S_t = \max\left(0, S_{t-1} + \left(\frac{n_t - (\mu_t + k\sigma_t)}{\sigma_t}\right)\right)$$

where k is a hyperparameter, μ_t and σ_t are as defined above, and S_{t-1} is the algorithm statistic for the previous time period. The UCL is defined as a constant h. We leverage a modified version of the EARS C3 algorithm, which monitors the sum of positive deviations in the current and previous two time periods. Whereas the traditional EARS C3 measures daily counts, here we aggregate counts by week and use an 8-week sliding baseline.

EWMA and CUSUM are best suited for detecting cumulate deviations from the baseline whereas X-bar is best suited for the detection of point deviations. Because we are aggregating cases by weeks, and it is unlikely that an outbreak will last more than 1 week, X-bar is likely to perform better. (Note if we didn't aggregate by week, EWMA or CUSUM may be better). When two consecutive weeks have high case counts, X-bar only counts that as one outbreak while CUSUM and EWMA are more likely to count them as two outbreaks. According to the true outbreak data, only one of those weeks is a "true outbreak," which is consistent with the duration of non-bloody diarrheal disease outbreaks among military populations of similar size and similar settings. Therefore, X-bar is better suited for this type of data.

Supplemental Data Evaluating an Alternate Cutoff for Inclusion of Data from a Given Epidemiologic Week

Weeks of epidemiologic silence where there is no reporting are difficult to differentiate from weeks with zero ADD cases. In light of this, we simultaneously ran all outbreak detection algorithms only on the weeks where we knew there was no epidemiologic silence. That is, weeks with at least one reported ADD case. In this setting, X-bar produced 13 signals, EWMA produced 22, and CUSUM produced 21 signals. Algorithm specificity across the five sites was

98.6% for X-bar, 96.9% for EWMA, and 97.1% for CUSUM. PPV was 46.2% for X-bar, 27.3% for EWMA, and 28.6% for CUSUM. There was a significant difference between the X-bar chart and EWMA algorithms in this setting.

References

1. Fricker R. Introduction to statistical methods for biosurveillance: with an emphasis on syndromic surveillance. Cambridge: Cambridge University Press; 2013. p. 178–215.

2. Burkom H. Development, adaptation, and assessment of alerting algorithms for biosurveillance. Johns Hopkins APL Tech Dig. 2003;24:335–42 [cited 2020 Apr 4]. https://www.jhuapl.edu/content/techdigest/pdf/v24-n04/24-04-burkom.pdf

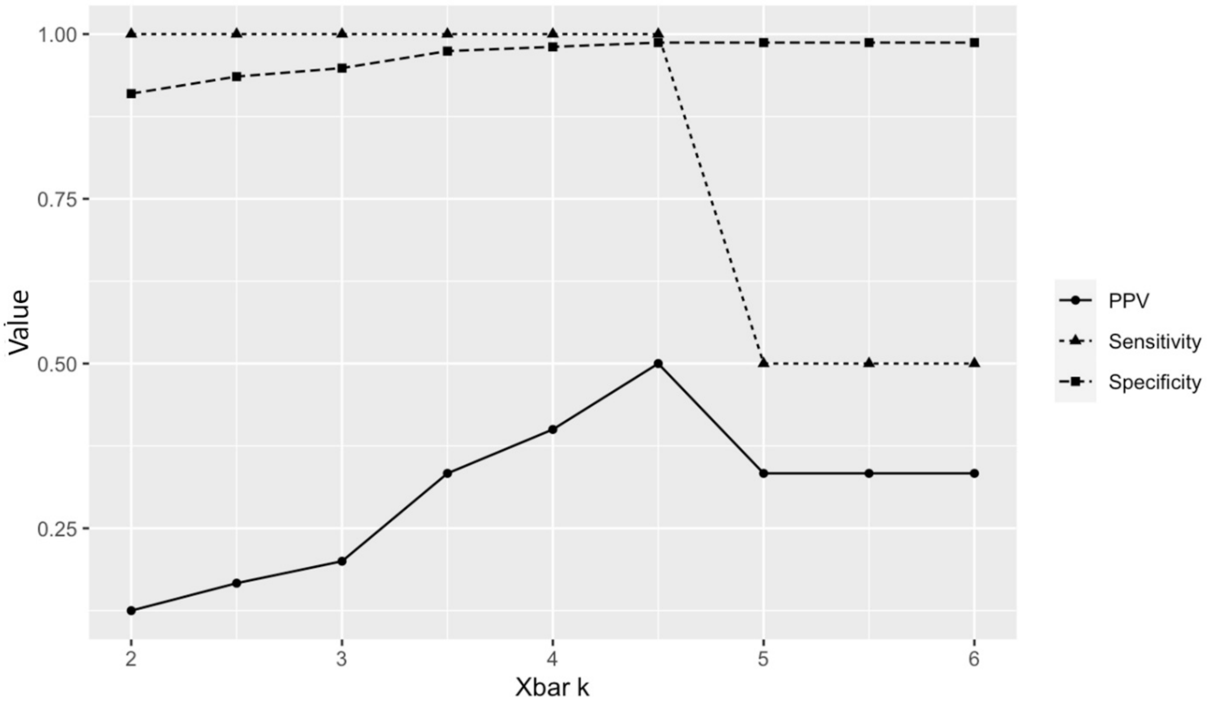

Appendix Figure 1. Sensitivity analysis demonstrating the effect of X-bar k on the model's sensitivity, specificity, and PPV for a randomly selected base, Policlínico Naval Ancón.

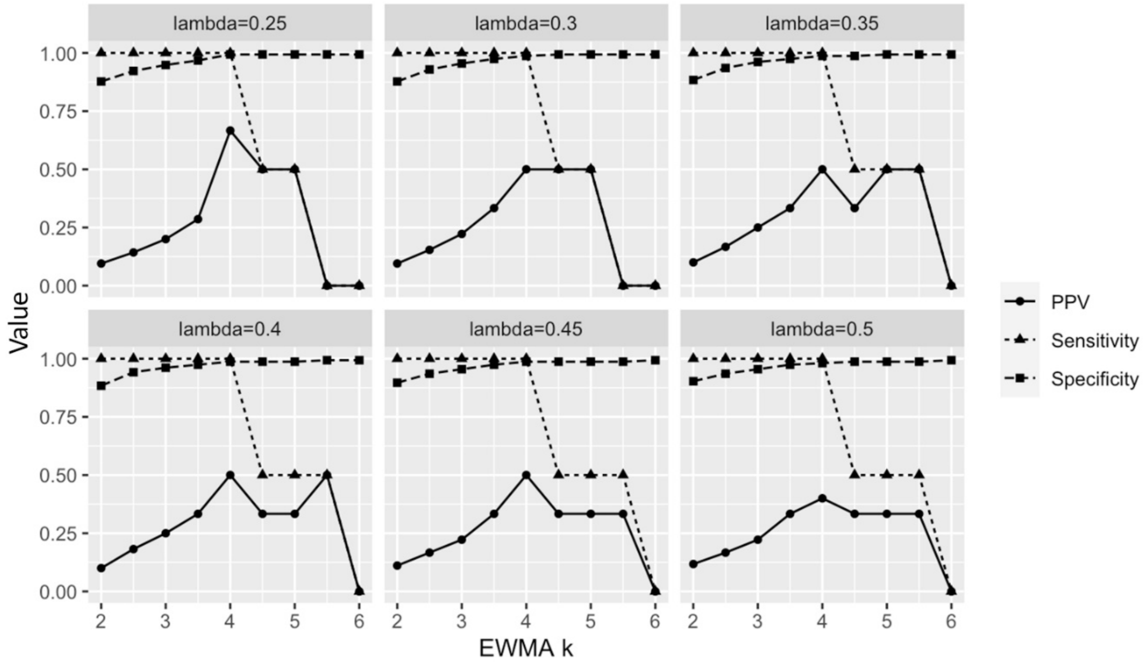

Appendix Figure 2. Sensitivity analysis demonstrating the effect of EWMA k and λ on the model's sensitivity, specificity, and PPV for a randomly selected base, Policlínico Naval Ancón.

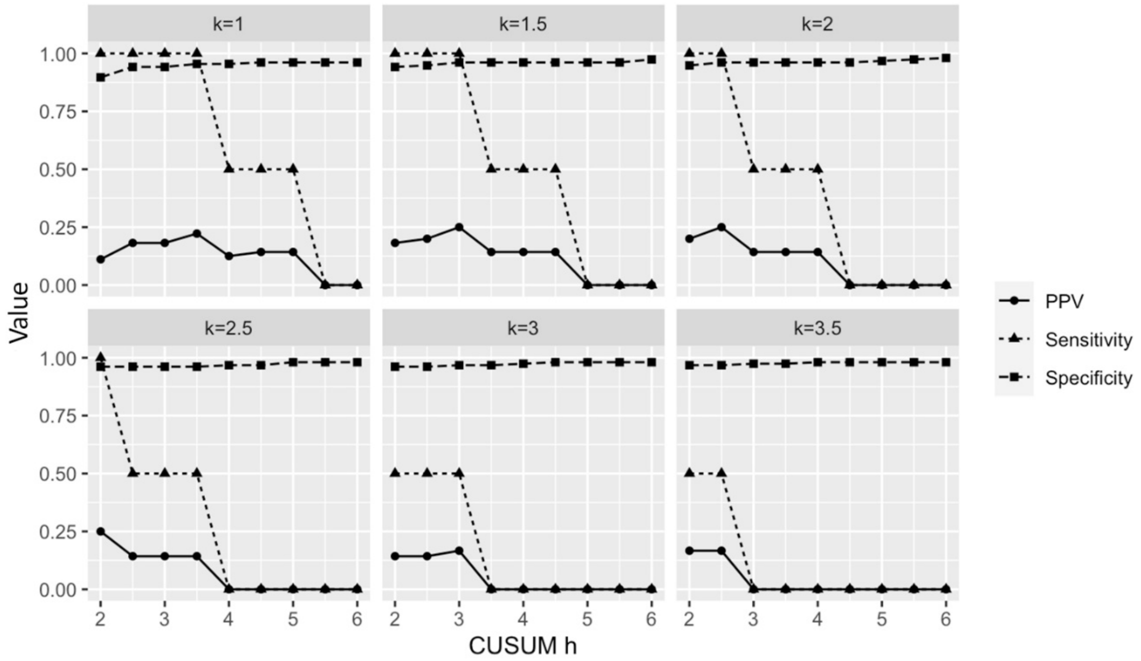

Appendix Figure 3. Sensitivity analysis demonstrating the effect of CUSUM k and h on the model's sensitivity, specificity, and PPV for a randomly selected base, Policlínico Naval Ancón.
